# What Hypodermatic Tablets Ought to Be

**Published:** 1899-01

**Authors:** 


### SELECTIONS.

### What Hypodermatic Tablets Ought To Be.

Absolutely correct in dose.

Well-formed and firm enough to resist ordinary handling.

Rapidly soluble.

Absolutely pure and irreproachable, in respect to their ingredients.

Prepared with a diluent incapable of doing the slightest mischief.

All hypodermatic tablets *ought* to possess these features—our tablets *do* possess them.—*Therapeutic Notes.*

